# Potential Application of MicroRNAs and Some Other Molecular Biomarkers in Alzheimer’s Disease

**DOI:** 10.3390/cimb46060304

**Published:** 2024-05-22

**Authors:** Olga Paprzycka, Jan Wieczorek, Ilona Nowak, Marcel Madej, Barbara Strzalka-Mrozik

**Affiliations:** 1 Department of Molecular Biology, Faculty of Pharmaceutical Sciences in Sosnowiec, Medical University of Silesia, 40-055 Katowice, Poland; s78207@365.sum.edu.pl (O.P.); s78223@365.sum.edu.pl (J.W.); mmarcel281297@gmail.com (M.M.); 2 Silesia LabMed, Centre for Research and Implementation, Medical University of Silesia, 40-752 Katowice, Poland; mc.ilona.nowak@gmail.com

**Keywords:** Alzheimer’s disease, neurodegeneration, miRNA, biomarkers, genes, molecular diagnostics, molecular mechanisms, amyloid beta

## Abstract

Alzheimer’s disease (AD) is the world’s most common neurodegenerative disease, expected to affect up to one-third of the elderly population in the near future. Among the major challenges in combating AD are the inability to reverse the damage caused by the disease, expensive diagnostic tools, and the lack of specific markers for the early detection of AD. This paper highlights promising research directions for molecular markers in AD diagnosis, including the diagnostic potential of microRNAs. The latest molecular methods for diagnosing AD are discussed, with particular emphasis on diagnostic techniques prior to the appearance of full AD symptoms and markers detectable in human body fluids. A collection of recent studies demonstrates the promising potential of molecular methods in AD diagnosis, using miRNAs as biomarkers. Up- or downregulation in neurodegenerative diseases may not only provide a new diagnostic tool but also serve as a marker for differentiating neurodegenerative diseases. However, further research in this direction is needed.

## 1. Introduction

Alzheimer’s disease (AD) is one of the most commonly occurring neurodegenerative diseases, although its etiology is not entirely understood. The disease is associated with the appearance of toxic β-amyloid (Aβ) and the accumulation of tau protein tangles within neurons, dysfunction in glucose metabolism, and improperly functioning mitochondria [1,2,3]. AD leads to the loss of neurons and gradual memory loss, cognitive dysfunction, behavioral changes, and eventual death. It is estimated that 44 million people worldwide suffer from the disease, a number that can double every 20 years [1,4]. The risk of developing AD increases with age, reaching approximately 30% at the age of 85 [4].

The disease can be divided into three stages: prodromal or pre-symptomatic, lightly symptomatic, and clinical AD [1]. Alzheimer’s disease has two main variations: early-onset Alzheimer’s (EOAD) and late-onset Alzheimer’s (LOAD). EOAD is hereditary, passed on genetically with an autosomal dominant pattern, and its symptoms begin before 65 years of age. Changes occur in the *PSEN1*, *PSEN2*, and *APP* genes [5]. LOAD typically develops after the age of 65, with its main risk factor being age. Various factors contributing to LOAD include sex, depression, genetic vulnerability, and social isolation [6,7].

AD manifests through gradual memory loss, disturbances in cognitive ability, and behavioral changes. These symptoms are nonspecific and are recognized via neurological testing [4]. Main methods of AD diagnosis utilize cerebrospinal fluid (CSF) analysis and positron emission tomography (PET) scans [8]. In CSF analysis, the total concentration of tau, p-tau, and Aβ proteins is ascertained with a specificity of 85–90%. Meanwhile, a PET scan shows nearly 100% specificity and 96% effectiveness. Both PET scans and CSF analysis reveal that disease progression can begin as early as 20 years before the onset of the first symptoms of AD [9].

The current standard for diagnosing AD in many countries relies on the diagnostic criteria outlined in the DSM-5. This approach is symptomatic and primarily based on the patient’s medical history rather than strictly measurable biomarkers. Diagnosis using this method relies on the appearance and progression of cognitive symptoms over time [10,11,12]. The method often yields accurate diagnoses, but it has limitations. Notably, the presence of significant cognitive impairment typically indicates advanced disease progression. Additionally, patients with extensive cognitive impairment may face challenges in undergoing diagnostic procedures or treatment due to the severity of their symptoms [10,11,12]. Although diagnostic methods such as positron emission tomography (PET) are highly accurate and advanced for early-onset AD (EOAD) or atypical cases, the limitations of the current diagnostic approach underscore the importance of discovering methods based on quantifiable protein indicators. Particularly promising are methods that are easy to administer and detectable before the onset of full clinical symptoms [10,11,12].

The main goal of AD treatment strategies is to inhibit the progression of the disease. Currently, four drugs are available in the pharmaceutical industry, belonging to two categories: anticholinesterase inhibitors (donepezil and galantamine) and drugs with antiglutaminergic effects (rivastigmine and memantine) [13,14,15]. Patients with AD typically have reduced levels of acetylcholine, a neurotransmitter responsible for transmitting information between nerve cells. Anticholinesterase inhibitors are used to increase acetylcholine levels, while antiglutaminergic drugs lower glutamate levels, which, if elevated, can lead to neuronal death. These drugs are administered orally or transdermally with the aim of slowing disease progression and stabilizing or improving patient functioning [16]. However, it is important to note that these medications do not address the underlying causes of AD. Furthermore, recent agents classified for preclinical and clinical trials have been unsuccessful, with a major limitation being the blood–brain barrier’s hindrance to drug transport into cells [9].

Due to the pathological processes that initiate long before the onset of symptoms and the limited treatment options available, predictive biomarkers for AD are actively being sought.

Currently, research is underway on methods to measure the levels of the two major forms of β-amyloid (Aβ40 and Aβ42) and phosphorylated tau protein in blood serum [17,18,19]. A major challenge in detecting such biomarkers is their very low concentration in blood compared to their concentrations in CSF. Another potential biomarker for AD is neurogranin, a calmodulin-binding protein [20,21]. Neurogranin, a postsynaptic protein influencing long-term synaptic potentiation (LTP), has been identified as relevant to AD. One study investigating the relationship between neurogranin and Alzheimer’s disease utilized data from the Alzheimer’s Disease Neuroimaging Initiative (ADNI) database. The study included patients without cognitive impairment as well as those with mild cognitive impairment and compared data over an eight-year follow-up period. Increased expression of neurogranin was observed in brain regions affected by AD [1,9]. The study demonstrated a significant increase in neurogranin levels in the CSF of Alzheimer’s patients. Moreover, this increase was not observed in other neurodegenerative diseases [22].

Another biomarker protein associated with AD is the cytoskeletal protein neurofilament light protein (NFL), known as a non-specific marker of neuronal damage [23,24]. A study investigating NFL protein also utilized the ADNI database, including patients with AD, individuals with mild cognitive impairment, and healthy subjects. NFL levels have been found to correlate with cognitive decline and can be measured in both serum and CSF [1,9,25].

Preische et al.‘s study [26], which utilized the Dominantly Inherited Alzheimer Network (DIAN) database, included subjects carrying mutations in the β-amyloid precursor protein genes, presenilin 1, or presenilin 2, along with a control group [26]. This study demonstrated that NFL can effectively distinguish between the hereditary and sporadic forms of AD. However, a limitation of NFL as a biomarker is that its levels also increase in other neurodegenerative diseases [1,9,26].

Another avenue of AD research involves assessing the potential of microRNAs (miRNAs) in its diagnosis [27]. miRNAs are small single-stranded RNA molecules that bind to specific mRNAs, leading to their inhibition or degradation [27]. It has been demonstrated that miRNAs play a role in pathological processes associated with AD, including the phosphorylation of tau protein and the formation of amyloid-beta precursor protein (APP) and amyloid-beta (Aβ) [1,9,27]. These molecules offer many diagnostic advantages, including their broad availability and stability in body fluids. Their bioavailability is related to both their small size and their binding to the Ago2 protein. MiRNAs bound to Ago2 exhibit particularly high stability and thus present the greatest potential as biomarkers [28]. Furthermore, when combined with Ago2 proteins, they form complex structures that further enhance their resistance to solubilization and increase their overall stability. Importantly, the association of these RNAs with proteins facilitates their detection and prolongs their persistence in the blood or cerebrospinal fluid before degradation [28]. The stability of miRNAs varies depending on the type of miRNA, biological material, and storage conditions [29]. Balzano et al. [29] conducted a study demonstrating that blood samples stored at −80 °C for several years did not undergo significant degradation of miRNA molecules [29]. Conversely, Sethi and Lukiw [30] investigated the stability of miRNA-9 in brain sections and nerve cells, finding respective half-lives of 0.9 h and 0.7 h [30]. Additionally, in cell lines, the stability was observed to be 90 min [30]. These findings suggest that the time elapsed from sample collection to freezing is critical, given the rapid degradation rate of miRNAs in biological samples. Moreover, it has been shown that levels of specific miRNAs differentiate between stages of the disease and can be used for the diagnosis of AD [1]. Additionally, currently researched potential markers for AD include nervous system inflammation markers, thyroid polypeptide, epidermal growth factor, lipid-based biomarkers, genetic biomarkers, kidney/brain protein (KIBRA), and neurotrophic brain-related factors [1,9].

Considering the increasing cases of AD diagnosis, the present review focuses on summarizing current information on AD biomarkers, with a particular emphasis on miRNAs as potential markers of the early detection of AD.

## 2. Molecular Mechanisms of Alzheimer’s Disease Pathogenesis

### 2.1. Role of β-Amyloid Precursor Protein (APP) and β-Amyloid

The Aβ protein appears to be the main etiological factor in the development of AD. The genes encoding it are located on chromosome 21 and consist of 18 exons. This means that the incidence of neurodegenerative diseases particularly affects people with Down syndrome, where the development of the disease begins at the age of 40 [31,32,33]. Additionally, genetic EOAD is associated with duplications of the *APP* gene on chromosome 21. However, partial trisomy 21 without duplication of the *APP* gene does not increase the chances of developing EOAD [31,32,33]. Increased expression via over-regulation in the *APP* gene promoter and mosaic amplification of this gene have been detected in cases of the disease. This is significant due to it suggesting that determining the amount of *APP* mRNA expression may be a useful tool for analyzing the progression and pathophysiology of AD [31,32,33].

The APP has 11 isoforms formed by alternative splicing, with the most common being APP695, APP751, and APP770 [31]. The protein has multiple functions; however, many of them are unclear and difficult to directly connect to AD. It exhibits low tissue specificity, with its expression being highest in the brain, although it is also expressed in many other tissues. At the cellular level, the protein is mainly expressed in the Golgi apparatus, at the cell membrane, and extracellularly. It interacts in complex ways with many other proteins [6,28,31,34].

The pathogenic processing of APP proteins occurs via the amyloidogenic pathway, which involves processing by BACE1 and γ-secretase enzymes. However, it also undergoes non-pathological proteolytic processing via α-secretase and γ-secretase [32,35,36]. In this pathway, the *N*-terminal fragment of the protein, which plays roles in proper brain function, is cleaved off. After undergoing the amyloidogenic pathway, the APP protein is fully converted into Aβ, and together with tau proteins, forms neurofibrillary tangles (NFT) structures, or neurotoxic fibrillar tangles of proteins that are considered a major pathogenic factor in both AD and other neurodegenerative diseases [32,35,36].

APP protein expression is regulated by its atypical promoter (which does not contain the traditional TATA or CAAT cassettes; instead, this function is assumed by region 5′ of the GGGCGC cassette) via transcription factors such as SP-1, activation protein AP-1, TGF-β, CTCF protein, heat shock protein (HSF-1), NF-κB, and androgens [32].

### 2.2. Tau Protein in Alzheimer’s Disease

The human tau protein is encoded by the *MAPT* gene, which is located on chromosome 17 and consists of 16 exons. As a result of alternative splicing, six isoforms of tau protein are produced, which differ in the presence or absence of 29 amino acid insertions at the *N*-terminus of the protein (RD region) [32,37,38]. These isoforms are labeled as 0N, 1N, 2N, 3R, and 4R, depending on the number of repeats. Healthy individuals have an equal expression ratio of 3R and 4R isoforms. However, an improper ratio can occur due to transcription errors, leading to diseases known as tauopathies [32,37,38,39].

The tau protein is primarily expressed in the brain with the highest levels found in the cerebral cortex. However, significant expression is also observed in muscles, kidneys, and the digestive system. At the cellular level, tau protein can be found in nuclear granules, but it is predominantly detected in the cell membrane [28,40,41]. Functionally, the protein plays a crucial role in stabilizing axonal microtubules in the brain, thereby facilitating transport within axons. The *N*-terminal region of the repeat domain (RD) is responsible for its aggregation. The neurotoxicity associated with tau protein and the NFTs containing it is likely linked to its propensity to aggregate [37,42].

The impact of the ratio of 3R and 4R proteins on the development of AD is not fully understood, as it is often multifactorial, challenging to analyze, and varies among patients. However, it appears that an excess of the 4R form promotes the production of NFT formations [30]. While it may not be a primary etiological factor in AD, its presence is significant in AD pathology. It is known that the development of AD is associated with the behavior of the tau protein, with pathology initiating in the hippocampus and intraparietal cortex before spreading to other regions of the brain [42,43].

### 2.3. Cellular Prion Protein in the Development of Alzheimer’s Disease

The prion protein (PrP) is present in almost all vertebrates at every stage of life and is expressed on the cell membranes of neurons throughout the brain [44]. What defines this protein as a prion is its remarkable ability to propagate its misfolded structure to other proteins of the same type. Essentially, a misfolded prion protein acts as an infectious agent, capable of replicating itself by inducing misfolding in other proteins—a process known as prion transformation [44]. This transformation is associated with severe and incurable neurodegenerative diseases, with Creutzfeldt–Jakob disease (CJD) being the primary prion disease in humans, characterized by rapidly progressive encephalopathy [44].

In AD, the PrP protein interacts with Aβ and oligomers, particularly those of high molecular weight [45,46]. These interactions impact neuronal responses to Aβ oligomers, however, do not appear to affect the neurotoxic effect of NFTs [31,45]. A study by Beraldo et al. [47] investigated the similarities in the interactions of misfolded prion proteins and β-amyloid with the prion protein–mGluR5 complex [47]. This complex has been identified as a crucial component of toxic pathways, particularly in prion disease. Evidence supporting this includes observations that mGluR5 null mice exhibit a significantly delayed onset of symptoms after prion infection compared to wild-type mice [47]. The binding of β-amyloid oligomers to this complex produces similar effects, as demonstrated by reduced toxicity in the absence of PrPc protein, mGluR5 protein, or both [47]. Interestingly, the absence of both PrP and mGluR5 proteins does not seem to have an additive effect, suggesting that they exhibit toxicity only when bound together rather than separately [47]. Notably, interference with mGluR5 expression has shown improved results in AD mouse models while exhibiting minor effects in prion-infected mice [47].

However, some studies have cast doubt on the direct involvement of the PrP protein as a toxic factor, suggesting that interactions between PrP and Aβ and tau proteins are not entirely conclusive [48]. In a study by Balducci et al. [48], it was demonstrated that when Aβ oligomers are directly injected into Prnp null neuron cells, their survival rate is comparable to that of Prnp-positive cells [48]. This finding, among others, implies that while the PrP protein does interact with Aβ oligomers, their toxicity may be independent of this interaction. Furthermore, it is suggested that the relationship between the prion protein and β-amyloids is bidirectional, which may complicate the interpretation of test results related to these interactions [48]. Nonetheless, this study confirms that the prion protein exhibits a high affinity for beta-amyloid oligomers, even though this affinity does not directly correlate with toxicity [48].

### 2.4. Role of Neurofibrillary Tangles in Alzheimer’s Disease

Neurofibrillary tangles are the primary pathological hallmark of AD, found within intraneuronal space and exerting a neurotoxic effect [43]. They predominantly consist of fragments of amyloid proteins and aggregates of tau proteins, including both 3R and 4R forms. NFT structures exhibit a β-helical-like structure. The process underlying their formation remains incompletely understood and controversial, but it is believed that they require amyloid granules to aggregate into full-sized NFT tangles [43]. Tau proteins present in NFTs possess prion-like properties and can transfer these properties to other tau proteins, contributing to tangle growth [43].

The pathological process may initiate due to sporadic mutations, mechanical damage, or inflammation, leading to the formation of granules that aggregate over time. Due to their structure, NFTs are resistant to attacks from kinases, proteases, and chaperones. The aggregates exert a chronically toxic effect [49,50]. Moreover, the granules around which the aggregates form can propagate from cell to cell via previously unknown mechanisms. Symptoms of AD typically manifest when NFTs are present in large quantities [49,50].

Theories regarding AD treatment suggest that preventing the proliferation of NFTs may offer the most effective approach to prevent disease onset and halt disease progression [49,50].

### 2.5. Role of Microbiome in Alzheimer’s Disease

**Figure 1:**
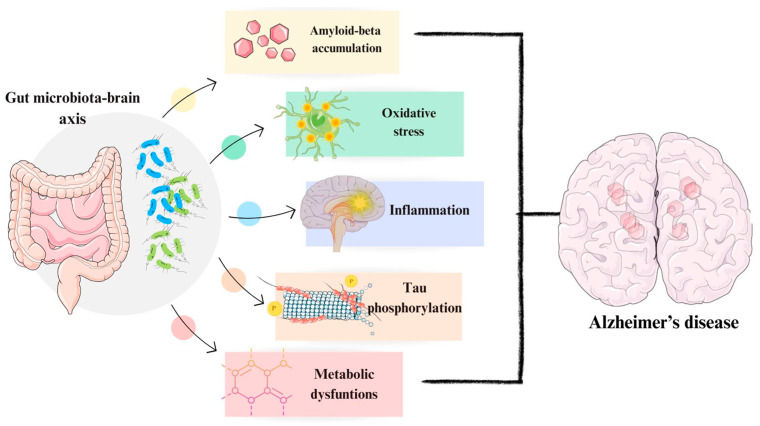
The role of the gut microbiota in Alzheimer’s disease [51]. The figure was partly generated using Servier Medical Art, provided by Servier and licensed under a Creative Commons Attribution 4.0 unported license.

In addition to the previously mentioned factors influencing the development of Alzheimer’s disease, the gut microbiota (GM), which mostly consists of bacteria (mainly *Acitinobacteria* and *Firmicutes*), but also taxa such as Archaea and Eukarya, plays an important role [51,52]. Its main task is to metabolize compounds, protecting against the penetration of pathogenic organisms into the intestine, but it also indirectly affects communication between the gastrointestinal tract and the nervous system [51,52]. The GM is also involved in neurotransmission, maintaining normal homeostasis of the body, and is responsible for regulating the development of neurons [51,52]. GM also plays a key role in Aβ accumulation, increased phosphorylation of tau protein, and is also involved in oxidative stress (Figure 1) [51,52].

The primary connection between the gastrointestinal tract and the nervous system is facilitated by the vagus nerve. Operating primarily within the parasympathetic nervous system, a division of the autonomic nervous system, the vagus nerve serves as the conduit for this connection [53,54]. Additionally, another mode of communication occurs via substances secreted by both the gut and its resident bacteria. These substances primarily include monoamines, amino acids, and short-chain fatty acids, which can traverse the blood–brain barrier and enter the brain [53,54]. The brain reciprocates communication with the gut directly via the vagus nerve and by releasing neurotransmitters that influence both the microbiome and gut function. One harmful mechanism by which the gut microbiome affects the brain is via the release of neurotoxic substances such as ammonia [53,54]. However, more relevant to Alzheimer’s disease is its ability to stimulate the immune system by secreting pro-inflammatory proteins like cytokines [53,54].

A specific type of microbiome deregulation studied in the context of AD is known as dysbiosis. Dysbiosis refers to a change in the composition of the gut microbiome, typically involving alterations in the presence and concentration of certain bacterial species in response to a disease. While certain forms of dysbiosis are unique to AD, they also correlate with dysbiosis associated with aging [53,55].

In a study conducted by Ferreiro et al. [56], a metagenomics analysis of patients with AD was performed to determine the composition of their microbiome compared to controls. The study revealed statistically significant differences between the two groups and identified a set of bacterial species specifically correlated with AD [56]. These species include *Oscillibacter* sp., *Faecalibacterium prausnitzii*, *Coprococcus catus*, and *Anaerostipes hadrus*. However, it is crucial to consider the bidirectional nature of the gut-brain connection when evaluating the impact of the microbiome on the brain [56]. This is particularly relevant for treatment strategies targeting AD and other neurological disorders that affect the gut. Due to the bidirectional nature of this connection, it is challenging to determine whether changes in the gut are a response to alterations in the brain or vice versa [56].

It appears that the most promising approach for microbiome analysis in the context of AD involves genomics studies of bacterial species [54,55]. This approach differs from traditional biomarker identification in blood or CSF and may serve as a valuable complementary tool for guiding brain analysis [56]. If specific bacterial species are identified as correlated with AD and preclinical AD, these findings could be utilized in a preliminary screening capacity [56]. They could serve as easily accessible indicators, directing patients toward more targeted screening and diagnosis methods [56].

## 3. Association of MicroRNAs with Alzheimer’s Disease

### 3.1. Influence of MicroRNAs on β-Amyloid Formation

MicroRNAs are small, non-coding nucleotide acids with an average length of 22–23 nucleotides. These molecules are transcribed in the cell nucleus into primary miRNAs (pri-miRNAs), which are subsequently processed by the enzyme Drosha [57]. The resulting 70-nucleotide precursors are then transported into the cytoplasm. Within the cytoplasm, the nucleic acid is further processed by the RNase III enzyme Dicer and binds to Agonaute (Ago) family proteins [57].

**Figure 2:**
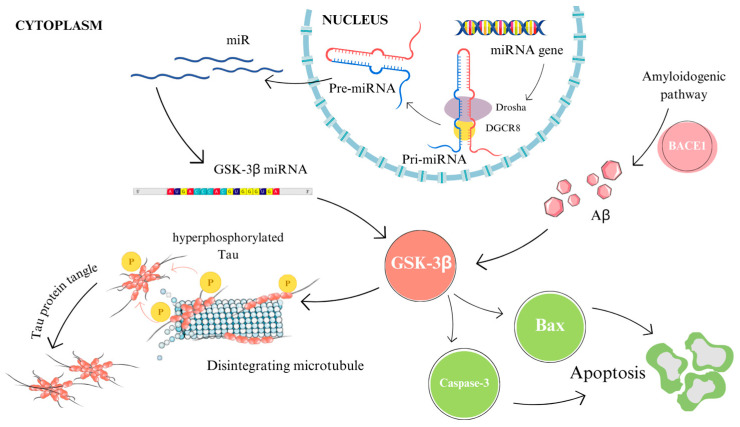
Mechanism of action of microRNAs in Alzheimer’s disease. The figure was partly generated using Servier Medical Art, provided by Servier and licensed under a Creative Commons Attribution 4.0 unported license.

MiRNAs possess the ability to bind target mRNAs, thereby blocking their translation or targeting them for degradation. Moreover, miRNAs that remain in complex with Ago proteins are stable and present in biological fluids (Figure 2) [57]. Their presence in exosomes is also significant, as the contents released from exosomes can impact the functions of recipient cells [58].

The roles of miRNAs in the pathogenesis of AD have been extensively investigated. Research has demonstrated that miRNAs are involved in regulating astrocytes, microglia, the cerebrovascular system, and synaptic abnormalities associated with tau protein and Aβ, among other functions [59].

In a study by Koh et al. [60], overexpression of miRNA-485-3p was observed in primary mouse neurons derived from C57BL/6I mice transduced with miRNA-485-3p. The study also investigated the therapeutic potential of antisense oligonucleotide (ASO) targeting miRNA-485-3p in a mouse model of AD (5XFAD and B6SJLF1 mice) by intraventricular injection of the tested ASO [60]. The overexpression of miRNA-485-3p was found to affect Aβ accumulation by inhibiting Aβ phagocytosis. Additionally, it was associated with the upregulation of inflammatory processes and abnormalities related to tau protein [60].

In another study utilizing a mouse model of AD (APPSwe/PS1dE9) and human embryonic kidney (HEK) cells, it was found that the expression levels of miRNA-331-3p and miR-9-5p increase as AD progresses [61]. The study investigated the effects of administering antagomirs targeting miRNA-331-3p and miR-9-5p. Both miRNAs were shown to regulate autophagy receptors, and their overexpression was associated with increased accumulation of Aβ [61].

In the study conducted by Jiang et al. [62], the focus was on investigating the association between miRNA-137-5p and the USP30 deubiquitinase, which plays a role in mitophagy. The research demonstrated that miRNA-137-5p contributes to alleviating symptoms of AD by downregulating USP30 levels in both mouse (C57BL/6J) and cell (SH-SY5Y) models [62]. In the study, mice were surgically sectioned and injected into the hippocampal area. The AD+mmu-agomiR-137-5p group received 1.5 µL of antagomir at a concentration of 100 µM, while the AD+mmu-agomiR-137-5p+USP30 group received 1.5 µL of antagomir at a concentration of 100 µM along with 2 µL of lentivirus. Furthermore, the researchers established a link between miRNA-137-5p and Aβ-associated neurotoxicity [62]. It was found that miRNA-137-5p mitigates this neurotoxicity by targeting USP30 [62,63].

In a study conducted by Wang et al. [64], utilizing human cell lines (HeLa, U373MG/U373, HMC3, SK-N-SH), as well as human postmortem brain tissues, an association between miR-20b-5p and AD biochemical pathways was demonstrated. HeLa cells were treated with an oligomer mimicking miR-20b or NCM at a concentration of 30 nM, while human glioblastoma multiforme cells and primary cultured human brain cells were treated with 100 nM [64]. Additionally, APP siRNA was administered to HeLa cells at a concentration of 20 nM and to primary human brain cells at 50 nM. All experiments in the study utilized antagomir or miR-20b inhibitor at a concentration of 100nM [64]. The study revealed that miR-20b-5p influences the regulation of APP and Aβ. Specifically, miR-20b-5p was identified as a negative regulator of the APP protein, impacting both developmental and protective brain functions. Furthermore, the study demonstrated that miR-20b-5p in primary cultures of human neurons disrupts cell calcium balance, as well as neurite and neuronal growth [64]. These findings suggest an effect of miRNAs on Aβ metabolic pathways in Alzheimer’s disease.

### 3.2. Relating MicroRNAs with Tau Protein

The research conducted by Zheng et al. [63] connected miR-135a-5p with synaptic dysfunction and memory impairment. In a mouse model of AD, the expression of miR-135a-5p is found to be downregulated. This downregulation leads to the activation of the Rho pathway, which is associated with adducin 1 and protein kinase 2, ultimately resulting in memory impairment and dendritic dysfunction [63]. The study utilized C57BL/6 and APP-PS1 mice, mouse N2a cells, and human 293T cells. Notably, the downregulation of miR-135a-5p is influenced by the overexpression of tau protein and the downregulation of expression of the transcription factor Fox3 [63]. The normal function of Fox3 is crucial for nervous system development [63]. This study sheds light on the intricate molecular mechanisms underlying synaptic dysfunction and memory impairment in AD.

**Figure 3:**
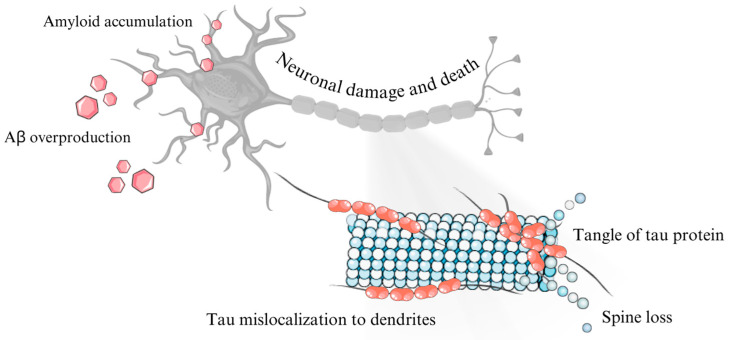
Role of tau protein in Alzheimer’s disease. The figure was partly generated using Servier Medical Art, provided by Servier and licensed under a Creative Commons Attribution 4.0 unported license.

In a study carried out by Nagaraj et al. [65], the protective effect of miR-483p in Alzheimer’s disease was demonstrated. The study utilized human HEK293, SK-N-MC, and HDFN cell lines. Cells were transfected with miRNA mimics at concentrations up to 100 nM along with GFP plasmid [65]. To induce Tau protein gene expression, HEK293 cells were transfected with 100 ng of pEGFP-TAU plasmid. Neonatal fibroblast cells designated for CRISPR/Cas9 editing were transfected with plasmid constructs. The experiment revealed that miR-20b-5p expression increases in patients with AD. Furthermore, miR-20b-5p was found to have a protective function by counteracting tau protein phosphorylation via regulation of the ERK1/ERK2 pathway [65]. These results suggest a significant association between miRNAs and the formation of defective tau protein. Additionally, miRNAs have the capability to counteract the formation of phosphorylated tau protein (Figure 3).

### 3.3. Involvement of MicroRNAs in Neurogenesis and Synaptic Plasticity

MicroRNAs play crucial roles in regulating various aspects of neurogenesis and synaptic plasticity in the brain by fine-tuning the expression of target genes involved in neuronal differentiation, maturation, and synaptic function [66].

In a study focusing on the role of miRNAs in AD, Walgrave et al. [66] demonstrated a decrease in miR-132 levels associated with Alzheimer’s disease. The experimental subjects included AD model mice, human neuronal progenitor cells, H9-GFP human embryonic stem cells, and HPC0A07/03C human multipotent progenitor/stem cells. To silence miR-132 gene expression, miR-132 antagomiR was injected intraventricularly into mice [66]. Conversely, if the miR-132 gene was overexpressed, mice were injected with miR-132 mimic or negative control oligonucleotides performed weekly for 4 weeks at a concentration of 150 pM. APPNL-GF mice were injected with gene construct viruses at concentrations of 10^9^ TU/mL for lentiviruses and 10^8 TU/mL for retroviruses, respectively [66]. The results and analysis of the conducted study revealed that miR-132 influences the ability to differentiate neurons, and its gene expression level is decreased in patients with AD. Moreover, in mouse models of AD with induced overexpression of the miR-132 , there was an increase in the level of cell proliferation and differentiation [66]. Furthermore, miR-132 was found to restore normal hippocampal neurogenesis in AD adults, leading to the restoration of memory deficits and reduction in cognitive impairment [66].

In a study by Liu et al. [67], an increase in miR-4722-5p gene expression in Alzheimer’s disease was observed, suggesting its impact on the pathogenesis and progression of the condition. The study conducted an analysis using RNA extracted from the serum of patients with AD, as well as PC12 rat tumor cell lines treated with Aβ_25__-__35_ [67]. The study demonstrated the overexpression of the miR-4722-5p in AD and identified genes involved in cyclic adenosine monophosphate (cAMP) degradation as its targets [67]. cAMP has been known to exert protective effects against AD, such as enhancing cognitive function, reducing memory deficits, and decreasing β-amyloid accumulation. Furthermore, the study identified the mTOR gene as one of the targets of miR-4722-5p. mTOR plays a role in autophagy-related processes, and dysfunction within mTOR pathways has been associated with neurodegenerative diseases [67]. These findings suggest the involvement of miRNAs in cell proliferation, differentiation, and autophagy in AD, highlighting their potential as key regulators of disease pathology.

## 4. Potential Molecular Markers of Alzheimer’s Disease

In Alzheimer’s disease, markers detected in CSF currently show the most promise, with many studies comparing their efficacy to established AD markers in CSF. However, it is important to acknowledge that collecting CSF can be challenging, particularly in elderly patients, and it requires a hospital setting for the procedure [68].

Another significant issue regarding AD markers is the disease’s frequent co-occurrence with other disorders. Therefore, for accurate diagnosis, a range of markers capable of distinguishing AD from other dementias is needed, along with markers specifically associated with AD [68].

Among markers detected in plasma, which are promising or being experimented with in specialized clinics, include the determination of Aβ_42/40_, phosphorylated tau, and plasma neurofilament light chain (NFL) levels [18]. The measurement of NFTs, Aβ, tau proteins, and their expression in nerve cells stands as the molecular marker with the most direct connection to the progression of Alzheimer’s disease [18].

The presence of Aβ in the brain serves as a crucial indicator for assessing the advancement of Alzheimer’s. However, a study conducted by Maltais et al. [69] suggested that the accumulation of Aβ in certain brain regions (such as the forebrain and cerebral cortex) correlates not only with Alzheimer’s disease but also with general aging processes of the brain [69]. While the study did not establish a direct link to AD, it suggests that meticulously examining β-amyloid accumulation in specific brain areas could potentially serve as a reliable method for predicting the pace of cerebral aging and the alterations that may lead to AD over time [69].

The PET method, such as the AMYPAD (Amyloid Imaging to Prevent Alzheimer’s Disease) technique, aims to quantify amyloid levels semi-quantitatively via PET scans [70]. This approach involves collecting extensive relative data, which undergoes computer processing to provide an assessment of amyloid levels in the brain without the need for invasive sampling procedures. According to Lopes et al. [70], this method is still undergoing further development. While it holds significant potential for diagnosing AD in the pre-symptomatic phase, current research directions associated with it have not fully explored its capabilities [70]. The method builds upon the existing practice of determining amyloid presence in the brain for AD diagnosis but introduces a novel semi-quantitative component. This innovation enables the method to be refined for diagnosing AD even before clinical symptoms manifest [70].

The study by Pereira et al. [71] demonstrated that a specific combination of P-tau_217_ and Aβ_42/40_ determination in plasma holds promise for diagnosing pre-dementia Alzheimer’s disease. These markers are detectable in a readily available fluid, with Aβ_42/40_ levels correlating with brain damage observed via PET scan methods [71]. P-tau_217_ (and to some extent p-tau_181_) is highly specific to Alzheimer’s disease and is not found in other forms of dementia, yet both markers emerge in the early stages of AD development. Combining these markers could offer an effective means of identifying preclinical Alzheimer’s disease and assessing a patient’s disease risk level [71].

In contrast, another study focusing on Aβ_42/40_ levels in plasma among a group of elderly individuals experiencing age-related issues did not find significant correlations with the presence of Aβ_42/40_. However, it did reveal that low plasma levels of this amyloid may be associated with an exacerbation of age-related problems in individuals with the APOE ε4 genotype [72].

The non-targeted metabolomics study conducted by Peña-Bautista et al. [73] identified potential markers for preclinical Alzheimer’s disease, including soraphene A, lyso PE, and resynamin. However, further experiments are necessary to confirm the usability of these markers in the early diagnosis of AD due to the preliminary nature of the study [73].

In another large-scale study investigating correlations between markers, researchers examined the presence of plasma lipids in the CSF of a sample comprising individuals with mild cognitive impairment, both related and unrelated to Alzheimer’s disease. The study observed reduced concentrations of sphingomyelins, particularly SM(d43:2) [74].

The study conducted by Ashton et al. [75] identified a significant challenge associated with using p-tau and beta-amyloid levels in plasma as diagnostic markers for AD. The research revealed that the content of p-tau increases significantly in the plasma of individuals who have experienced cardiac arrest, particularly immediately following the event and persisting for an extended period thereafter. In contrast, levels of NFL and t-tau remained stable [75]. Additionally, oligonucleotides of Aβ_40/42_ also increased 72 h post-cardiac arrest. These findings suggest that t-tau in plasma may offer a more promising avenue for research compared to p-tau, emphasizing the importance of investigating the stability of these markers across various conditions [75].

In turn, the study conducted by Gill et al. [76] highlights the potential challenges of using exosomal tau, Aβ_42_, and interleukin 10 as diagnostic markers for AD. Their study found an increased presence of these markers in soldiers with multiple head injuries, which is consistent with similar results observed in athletes playing sports prone to head injuries. Although the implications of these results for the diagnosis of AD are not definitive, they suggest that these markers may serve as general indicators of traumatic brain injury rather than being specific to AD [76]. However, an alternative interpretation of the findings is that head trauma serves as a risk factor for the development of AD. Repetitive trauma of this nature could potentially trigger the initiation of AD pathological processes, suggesting that the markers identified in individuals with head injuries may indeed be specific indicators of AD [76]. However, research in this area faces numerous challenges due to multifactorial influences, making it difficult to arrive at clear conclusions [77].

**Figure 4:**
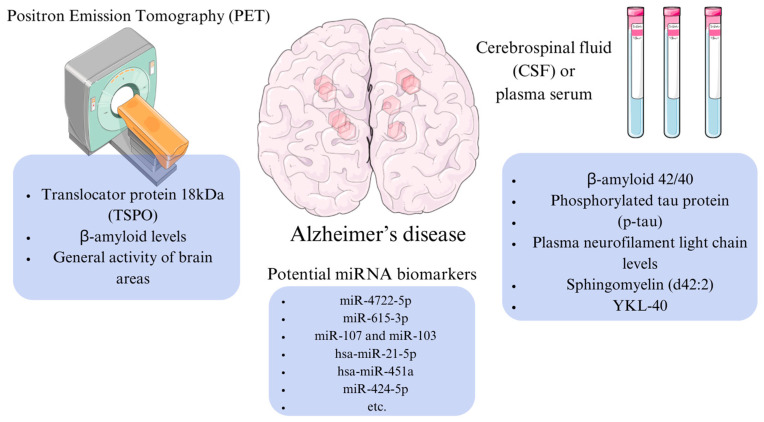
Alzheimer’s disease diagnostics methods and potential microRNA (miRNA) biomarkers. The figure was partly generated using Servier Medical Art, provided by Servier and licensed under a Creative Commons Attribution 4.0 unported license.

A study by Prins et al. [78] investigated the potential of inflammatory markers in diagnosing preclinical AD. The study suggested that the markers YKL-40 (CHI3L1), a glycoprotein derived from astrocytes, and GFAP (a marker related to astrogliosis) detected in plasma, could serve as effective indicators, particularly in pre-dementia patients [78]. This research aimed to assess the similarity of these markers to more established Alzheimer’s markers, such as Aβ_1-42_ in cerebrospinal fluid. The GFAP marker showed promise in predicting the severity of dementia progression later in life, while the YKL-40 marker could indicate the onset of Aβ-related pathological processes. It is worth noting that this study primarily examined correlations among markers themselves rather than directly linking them to pathological effects [78]. Key biomarkers in the diagnosis of AD disease are shown in Figure 4.

The study conducted by Chaney et al. [79] aimed to explore inflammatory microstates and their potential as markers for diagnosing AD. A key focus of the study was the assessment of translocator protein 18 kDa (TSPO) protein using PET scans. TSPO is a neuroinflammatory marker located near the outer mitochondrial membrane, and it is associated with inflammatory processes, neurosteroid production, and pathogenic mechanisms in various neurodegenerative diseases, including AD [79]. PET imaging targeting TSPO in the brain has shown promise as a method for diagnosing the stage of AD, albeit with limitations. These limitations include the use of older-generation tracers with suboptimal specificity and the non-specific nature of inflammatory neurostates for AD. While TSPO PET imaging may serve as a valuable tool for assessing disease progression, it is unlikely to be a standalone diagnostic method [79]. Furthermore, there remains a need for a better understanding of the relationship between neuroinflammation and the progression of neurodegenerative diseases, as well as the broader context of inflammatory microstates in the brain environment [79]. As knowledge in these areas advances, methods for evaluating neuroinflammation are expected to become more accurate and clinically useful [79].

The study by El Idrissi et al. [80] conducted a bioinformatics analysis to identify proteins strongly associated with neuroinflammation in AD. They identified several key proteins that warrant further investigation in the context of neuroinflammation in AD, including CCL2, CXCL8, IL-10, TLR4, and CRP. Additionally, they highlighted AKT1 as crucial for cellular information transfer, while IL4, IL10, and IL13 were suggested as potential secondary therapeutic targets for AD treatment [80].

On the other hand, Carlini et al. [81] investigated the cellular localization and abundance of the intercellular chloride channel 1 (CLIC1) protein as a marker for neurodegeneration. This protein tends to accumulate in peripheral blood mononuclear cells. Under normal physiological conditions, CLIC1 is a hydrophilic protein found in the cytoplasm [81]. However, chronic oxidative stress can lead to its translocation to the cell membrane. The localization of CLIC1 has been associated with chronic inflammation in the brain and the activation of microglia, indicating its potential role in neurodegenerative processes [81]. They revealed that the levels of membrane-bound CLIC1 are significantly higher in patients with AD compared to controls [81]. This novel finding suggests a potential diagnostic approach for AD, although the study focused on patients already diagnosed with clinical AD. Further research involving diverse populations and investigating the correlation between CLIC1 levels and other biomarkers could enhance the technique’s diagnostic utility [81].

In a study conducted by Kenny et al. [82], proteomic analysis of tear fluid collected from patients with AD identified significant differences in protein composition compared to controls. Among the most variable proteins were eIF4E and tropomyosin alpha 4. Additionally, altered levels of miRNAs were detected in tears from patients with AD compared to controls, indicating the potential of tear fluid analysis as a diagnostic tool for AD [82]. Furthermore, a study focusing on non-coding RNAs other than miRNAs revealed that the concentration of BACE1-related long non-coding RNAs (lncRNAs) excreted from cells in exosomes may offer another promising direction for AD diagnosis based on nucleic acids [83].

## 5. Application of microRNAs as Biomarkers for Alzheimer’s Disease Diagnostics

miRNAs hold great promise as non-invasive and reliable biomarkers for AD diagnostics. Continued research efforts aimed at identifying and validating specific miRNA signatures associated with AD pathology are crucial for advancing the field toward the development of effective diagnostic tools and personalized therapeutic strategies.

In a study conducted by Nie et al. [84], miR-424-5p was identified as a potential molecular marker for AD, alongside let-7e-5p, which could serve as a distinguishing molecule for various neurodegenerative diseases. The study utilized plasma exosomes obtained from patients with AD and Parkinson’s disease (PD), as well as from healthy controls [84]. Among the participants, there were 34 healthy subjects, 5 patients with AD, and 7 patients with PD. The findings revealed that the expression level of miR-424-5p was elevated in patients with AD but reduced in those with PD [84]. This particular miRNA is involved in regulating genes associated with brain energy metabolism. Furthermore, let-7e-5p, belonging to the let-7 neurotoxic family, exhibited higher expression levels in patients with PD compared to patients with AD, suggesting its potential utility as a discriminatory marker between the two conditions [84].

A study by Visconte et al. [85] also demonstrated the potential usefulness of miRNAs derived from plasma exosomes. The study used extracted miRNAs from 11 patients with AD, 20 control patients, and 19 patients with mild cognitive impairment without AD. It was shown that the expression levels of seven miRNAs were higher in patients with AD than in controls miR106a-5p, miR-16-5p, miR-223-3p, miR-25-3p, miR-30b-5p, miR-16-5p, miR-92a-3p, and miR-451a. In addition, four miRNAs were selected of which the levels are increased in prodromal AD—miR16-5p, miR-25-3p, miR-92a-3p, and miR-451a [85]. Dysregulation of these miRNAs has been shown to affect numerous cellular processes, including the Wnt signaling pathway, intercellular connections, adhesion, glucose metabolism, ubiquitination, and cytoskeletal organization, among others. Furthermore, the study revealed that the targets of these miRNAs include genes such as *NOTCH2*, *BTG2*, or *KCNC4* [85]. The NOTCH2 protein plays a role in processes such as differentiation, proliferation, and apoptosis, while the BTG2 protein is involved in cell cycle regulation. The KCNC4 protein, on the other hand, is associated with chemical synaptic transmission. Studies have supported the hypothesis of their potential utility in the diagnosis of AD [85].

A meta-analysis conducted by Liu et al. [86] revealed a significant association between blood exosomes and both preclinical and clinical AD. The analysis identified specific miRNAs, such as miR-132 and miR-212, derived from neurons, which demonstrated the ability to differentiate patients with AD from controls [86]. MiR-132 has been implicated in regulating synaptic plasticity by influencing steroid metabolism [87]. Furthermore, miR-384 has shown promise in distinguishing AD from other diseases presenting with cognitive impairment, such as vascular dementia and Parkinson’s disease. The target gene for miR-384 is gene encoding adaptor kinase 1, which is associated with neuronal damage and neurological diseases [87,88]. Notably, hsa-miR-21-5p and hsa-miR-451a have emerged as potential molecular markers for differentiating AD from dementia characterized by Lewy bodies [86]. These miRNAs are associated with the PI3K/AKT signaling pathway, which regulates cell apoptosis [89,90].

In the study conducted by Liu et al. [67], miR-4722-5p and miR-615-3p were identified as potential molecular markers of AD. Serum samples from the patients and a PC12 cell line treated with Aβ_25-35_ at 30 µM/L were utilized in the study [67]. MiRNA-4722-5p is implicated in the degradation of cAMP, which is associated with memory-related processes, as well as mTOR and neurotrophins. Conversely, miR-615-3p is linked to the FoxO signaling pathway, involved in cell apoptosis and autophagy, among other functions. The study revealed elevated mRNA expression levels of these miRNAs in both patient serum and the PC12 cell line [67].

In another study by Wang et al. [91], the diagnostic potential of miR-103 and miR-107 was demonstrated. The research included patients with AD, patients with PD, and healthy individuals. Plasma samples were used to extract miRNAs for analysis. MiR-103 is involved in processes inhibiting cell apoptosis and axon growth, while miR-107 inhibits cell apoptosis and β-amyloid accumulation in AD [91]. MiR-103 exhibited the ability to differentiate between patients with AD and patients with PD as well as controls, whereas miR-107 could distinguish patients with AD from controls but not from patients with PD [91].

In summary, the study highlighted the diagnostic potential of the mentioned miRNAs and identified miR-103 as a more promising diagnostic marker compared to miR-107 [91]. Additionally, in a research paper by Souza et al. [92], miR-9 was singled out as a potential specific marker for Alzheimer’s disease. The study involved 74 participants, including 38 controls and 36 patients with probable AD. Whole blood was used as the biological material for miRNA extraction [92]. In humans, miR-9 influences the regulation of postmitotic neuronal differentiation and is implicated in the accumulation of Aβ or the elevation of Tau protein phosphorylation levels. The study demonstrated that the expression level of miR-9 in patients with AD was lower compared to the control group, suggesting its potential as a diagnostic biomarker for AD [92]. Other connections between miRNAs and AD are summarized in Table 1.

In summary, miRNAs exhibit the potential to modulate the onset and progression of Alzheimer’s disease by regulating the molecular mechanisms involved in AD pathogenesis. Therefore, they represent promising candidates for therapeutic interventions and diagnostic applications in AD.

## 6. Conclusions

Recent studies have strongly implicated miRNAs in the pathogenesis of Alzheimer’s disease. Dysregulated miRNA expression patterns have been observed in patients with AD, correlating with disease progression and severity. Profiling miRNA levels in biological fluids like blood or cerebrospinal fluid shows promise for identifying AD biomarkers, aiding in early diagnosis, prognosis, and disease progression monitoring. Additionally, miRNAs may help distinguish between different neurodegenerative diseases, each potentially exhibiting a unique miRNA signature reflective of its underlying pathology.

This review underscores the potential utility of miRNAs and other molecular biomarkers as diagnostic tools for Alzheimer’s disease. MiRNAs represent an AD-associated biological material that is readily available and stable in biological fluids, including blood. Moreover, the extraction procedure for miRNAs is relatively inexpensive and straightforward.

In conclusion, miRNAs hold promise as potential molecular markers of AD. However, further research and validation studies are needed to fully exploit their diagnostic and prognostic utility in the context of Alzheimer’s disease and related conditions.

## Figures and Tables

**Table 1:** Associations of microRNAs with Alzheimer’s disease.

miRNA	Function	Biological Material	Ref.
miRNA-101a	Negative modulation of autophagy	Human blood plasma, APPSwe/PS1ΔEP transgenic mice, Human SH-SY5U immature neuroblastoma cells	[93]
miRNA-128	Inhibition of tau protein phosphorylation and β-amyloid accumulation	Human cerebral cortex tissues from patients with AD and control patients, mouse Neuro-2a neuroblast cells, human SH-SY5Y immature neuroblastoma cells, transformed human embryonic kidney cells, HEK-293T	[94]
miRNA-146a	Pro-inflammatory function	Rat brain tissue (culture of glial and cortical neurons), Mouse hippocampal neuronal cell line, HT-22 cells, mouse cerebellar microglia, mouse vascular endothelial cells, mouse C8-B4 cells, mouse bEnd cells.3; Human brain tissue of the frontal cortex, temporal cortex, and cerebellum of patients with AD and controls	[95]
miRNA-29c-3p	Negative regulation of BACE1, a key enzyme that degrades amyloid precursor protein	SPF C57BL/6J mice, PC12 cell line	[96]
miRNA-130a	Protective function resulting from targeting *DAPK1*, a serine/threonine kinase gene associated with neuronal apoptosis	SH-SY5Y cell line, AD APPSwe/PS1dE9 mice, wild-type mice	[97]
miRNA-451a	Protective function, inhibition of BACE1, inhibition of inflammatory processes by negative regulation of NLRP3	Human cerebrospinal fluid, APP/PS1 transgenic mice, wild-type mice, mouse line Neuro-2a, human line HEK293,	[98]
miRNA-485-3p	Accumulation of β-amyloid, pathologies of tau protein, development of the inflammatory process of the nervous system	Human frontal cortex, medial cortex, and CSF samples from AD and control patients, human plasma samples, B6SJLF1/J mice, 5XFAD transgenic mice, C57BL/6 mice	[60]
miRNA-126, miRNA-135	Protective function related to the removal of β-amyloid	Mice 3xTG-AD, B6129SF2 control strain	[99]
miRNA-181a	Toxicity associated with β-amyloid, a negative regulator of synaptic plasticity	Brain tissue and cell cultures, C57BL/J, 3xTg-AD mice, 129/C57BL6 hybrid control mice	[100]
miRNA-31	Protective function associated with targeting *APP* and *BACE1* genes	Mice 3xTG-AD, mouse line HT-22, human line HEK293, human line SH-SY5Y	[101]
miRNA-369	Phosphorylation of tau protein	miRNA-369 KO 3xTG-AD mice, C57/B6 mice, 3xTG-AD mice, 293T cells	[102]
miRNA-129	Modulation of *MAPK1* and *ERK* genes	Wistar rats	[103]
miRNA-34c	Modulation of the *SYT1* gene	HT-22 cell line, line 293a	[104]
miRNA-455-5p	*CPEB* gene regulation	APP/PS1 mice, wild-type control mice	[105]
miRNA-193b	The impact of the decrease in APP	Blood from patients with Alzheimer’s dementia, mild cognitive impairment, and subjective cognitive decline, patients as controls, APP/PS1 double transgenic mice, wild-type mice, mouse HT-22 line	[106]
miRNA-17	Downregulation of autophagy-related genes	Brain tissues of the human temporal lobe of patients with AD and controls, C57BL/6 wild-type mice, 5xFAD mice, Atg 5^−/−^ mice	[103]
miRNA-384	Decreased expression of genes encoding APP and β-secretase	Blood and CSF of patients with dementia due to AD, with mild cognitive impairment and subjective cognitive decline, control patients	[107]
miRNA-26b	Increase in β-amyloid production mediated by IGF1 regulation	n/d	[108]
miRNA-125b	Targeting 15-lipoxygenase, synapsin, complement factor H, tetraspanin 7 and tetraspanin 12	n/d	[109]

miRNA—microRNA; AD—Alzheimer’s disease; BACE1—beta-site APP cleaving enzyme; MAPK1—mitogen-activated protein kinase 1; ERK—extracellular signal-regulated kinase; DAPK1—death-associated protein kinase 1; NLRP3—NLR family pyrin domain containing 3; APP —amyloid beta precursor protein; SYT1—synaptotagmin 1; CPEB—cytoplasmic polyadenylation element binding protein 1; CSF—cerebrospinal fluid; IGF1—insulin-like growth factor 1; n/d—no data.

## Data Availability

Available upon request and with regulations.

## References

[1] Klyucherev T.O., Olszewski P., Shalimova A.A., Chubarev V.N., Tarasov V.V., Attwood M.M., Syvänen S., Schiöth H.B. (2022). Advances in the development of new biomarkers for Alzheimer’s disease. Transl. Neurodegener..

[2] Rostagno A.A. (2022). Pathogenesis of Alzheimer’s disease. Int. J. Mol. Sci..

[3] John A., Reddy P.H. (2021). Synaptic basis of Alzheimer’s disease: Focus on synaptic amyloid beta, p-tau and nitochondria. Ageing Res. Rev..

[4] Nikolac Perkovic M., Videtic Paska A., Konjevod M., Kouter K., Svob Strac D., Nedic Erjavec G., Pivac N. (2021). Epigenetics of Alzheimer’s disease. Biomolecules.

[5] Bertram L., Tanzi R.E. (2020). Genomic mechanisms in Alzheimer’s disease. Brain Pathol..

[6] Andrade-Guerrero J., Santiago-Balmaseda A., Jeronimo-Aguilar P., Vargas-Rodríguez I., Cadena-Suárez A.R., Sánchez-Garibay C., Pozo-Molina G., Méndez-Catalá C.F., Cardenas-Aguayo M.-C., Diaz-Cintra S. (2023). Alzheimer’s disease: An updated overview of its genetics. Int. J. Mol. Sci..

[7] Silva M.V.F., Loures C.d.M.G., Alves L.C.V., de Souza L.C., Borges K.B.G., Carvalho M.d.G. (2019). Alzheimer’s disease: Risk factors and potentially protective measures. J. Biomed. Sci..

[8] Parashat T., Sumathi V. (2023). Identification of Alzheimer’s disease by imaging: A comprehensive review. Int. J. Environ. Res. Public Health.

[9] Khan S., Barve K.H., Kumar M.S. (2020). Recent advancements in pathogenesis, diagnostics and treatment of Alzheimer’s disease. Curr. Neuropharmacol..

[10] Atri A. (2019). The Alzheimer’s disease clinical spectrum: Diagnosis and management. Med. Clin. N. Am..

[11] Porsteinsson A.P., Isaacson R.S., Knox S., Sabbagh M.N., Rubino I. (2021). Diagnosis of early Alzheimer’s disease: Clinical practice in 2021. J. Prev. Alzheimers Dis..

[12] Graff-Radford J., Yong K.X.X., Apostolova L.G., Bouwman F.H., Carrillo M., Dickerson B.C., Rabinovici G.D., Schott J.M., Jones D.T., Murray M.E. (2021). New insights into atypical Alzheimer’s disease in the era of biomarkers. Lancet Neurol..

[13] Knapskog A.-B., Engedal K., Selbæk G., Øksengård A.-R. (2021). Alzheimer’s disease—Diagnosis and treatment. Tidsskr Nor Legeforen.

[14] Balázs N., Bereczki D., Kovács T. (2021). Cholinesterase inhibitors and memantine for the treatment of Alzheimer and non-Alzheimer dementias. Ideggyogyaszati Szle.

[15] Athar T., Al Balushi K., Khan S.A. (2021). Recent advances on drug development and emerging therapeutic agents for Alzheimer’s disease. Mol. Biol. Rep..

[16] Passeri E., Elkhoury K., Morsink M., Broersen K., Linder M., Tamayol A., Malaplate C., Yen F.T., Arab-Tehrany E. (2022). Alzheimer’s disease: Treatment strategies and their limitations. Int. J. Mol. Sci..

[17] Lloret A., Esteve D., Lloret M.-A., Cervera-Ferri A., Lopez B., Nepomuceno M., Monllor P. (2019). When does Alzheimer′s disease really start? The role of biomarkers. Int. J. Mol. Sci..

[18] Hansson O., Edelmayer R.M., Boxer A.L., Carrillo M.C., Mielke M.M., Rabinovici G.D., Salloway S., Sperling R., Zetterberg H., Teunissen C.E. (2022). The Alzheimer’s association appropriate use recommendations for blood biomarkers in Alzheimer’s disease. Alzheimers Dement..

[19] Thijssen E.H., La Joie R., Strom A., Fonseca C., Iaccarino L., Wolf A., Spina S., Allen I.E., Cobigo Y., Heuer H. (2021). Plasma phosphorylated tau 217 and phosphorylated tau 181 as biomarkers in Alzheimer’s disease and frontotemporal lobar degeneration: A retrospective diagnostic performance study. Lancet Neurol..

[20] Saunders T., Gunn C., Blennow K., Kvartsberg H., Zetterberg H., Shenkin S.D., Cox S.R., Deary I.J., Smith C., King D. (2023). Neurogranin in Alzheimer’s disease and ageing: A human post-mortem study. Neurobiol. Dis..

[21] Chang C.-H., Lin C.-H., Lane H.-Y. (2021). Machine learning and novel biomarkers for the diagnosis of Alzheimer’s disease. Int. J. Mol. Sci..

[22] Headley A., De Leon-Benedetti A., Dong C., Levin B., Loewenstein D., Camargo C., Rundek T., Zetterberg H., Blennow K., Wright C.B. (2018). Alzheimer’s Disease Neuroimaging Initiative. Neurogranin as a predictor of memory and executive function decline in MCI patients. Neurology.

[23] Stevenson-Hoare J., Heslegrave A., Leonenko G., Fathalla D., Bellou E., Luckcuck L., Marshall R., Sims R., Morgan B.P., Hardy J. (2022). Plasma biomarkers and genetics in the diagnosis and prediction of Alzheimer’s disease. Brain.

[24] Leuzy A., Mattsson-Carlgren N., Palmqvist S., Janelidze S., Dage J.L., Hansson O. (2022). Blood-based biomarkers for Alzheimer’s disease. EMBO Mol. Med..

[25] Pereira J.B., Westman E., Hansson O. (2017). Alzheimer’s Disease Neuroimaging Initiative. Association between cerebrospinal fluid and plasma neurodegeneration biomarkers with brain atrophy in Alzheimer’s disease. Neurobiol. Aging.

[26] Preische O., Schultz S.A., Apel A., Kuhle J., Kaeser S.A., Barro C., Gräber S., Kuder-Buletta E., LaFougere C., Laske C. (2019). Serum neurofilament dynamics predicts neurodegeneration and clinical progression in presymptomatic Alzheimer’s disease. Nat. Med..

[27] Ying S.Y., Chang D.C., Lin S.L. (2008). The microRNA (miRNA): Overview of the RNA genes that modulate gene function. Mol. Biotechnol..

[28] Thul P.J., Lindskog C. (2018). The human protein atlas: A spatial map of the human proteome. Protein Sci..

[29] Balzano F., Deiana M., Dei Giudici S., Oggiano A., Baralla A., Pasella S., Mannu A., Pescatori M., Porcu B., Fanciulli G. (2015). miRNA stability in frozen plasma samples. Molecules.

[30] Sethi P., Lukiw W.J. (2009). Micro-RNA abundance and stability in human brain: Specific alterations in Alzheimer’s disease temporal lobe neocortex. Neurosci. Lett..

[31] Guo T., Zhang D., Zeng Y., Huang T.Y., Xu H., Zhao Y. (2020). Molecular and cellular mechanisms underlying the pathogenesis of Alzheimer’s disease. Mol. Neurodegener..

[32] Sato K., Takayama K., Hashimoto M., Inoue S. (2021). Transcriptional and post-transcriptional regulations of amyloid-β precursor protein (APP) mRNA. Front. Aging.

[33] Delport A., Hewer R. (2022). The amyloid precursor protein: A converging point in Alzheimer’s disease. Mol. Neurobiol..

[34] Dunot J., Ribera A., Pousinha P.A., Marie H. (2023). Spatiotemporal insights of APP function. Curr. Opin. Neurobiol..

[35] Sehar U., Rawat P., Reddy A.P., Kopel J., Reddy P.H. (2022). Amyloid beta in aging and Alzheimer’s disease. Int. J. Mol. Sci..

[36] Yarns B.C., Holiday K.A., Carlson D.M., Cosgrove C.K., Melrose R.J. (2022). Pathophysiology of Alzheimer’s disease. Psychiatr. Clin. N. Am..

[37] Tabeshmehr P., Eftekharpour E. (2023). Tau; one protein, so many diseases. Biology.

[38] Rawat P., Sehar U., Bisht J., Selman A., Culberson J., Reddy P.H. (2022). Phosphorylated tau in Alzheimer’s disease and other tauopathies. Int. J. Mol. Sci..

[39] Corsi A., Bombieri C., Valenti M.T., Romanelli M.G. (2022). Tau isoforms: Gaining insight into MAPT alternative splicing. Int. J. Mol. Sci..

[40] Antón-Fernández A., Vallés-Saiz L., Avila J., Hernández F. (2023). Neuronal nuclear tau and neurodegeneration. Neuroscience.

[41] Lester E., Van Alstyne M., McCann K.L., Reddy S., Cheng L.Y., Kuo J., Pratt J., Parker R. (2023). Cytosolic condensates rich in polyserine define subcellular sites of tau aggregation. Proc. Natl. Acad. Sci. USA.

[42] Wegmann S., Biernat J., Mandelkow E. (2021). A current view on tau protein phosphorylation in Alzheimer’s disease. Curr. Opin. Neurobiol..

[43] Jackson N.A., Guerrero-Muñoz M.J., Castillo-Carranza D.L. (2022). The prion-like transmission of tau oligomers via exosomes. Front. Aging Neurosci..

[44] Baldwin K.J., Correll C.M. (2019). Prion disease. Semin. Neurol..

[45] Panes J.D., Saavedra P., Pineda B., Escobar K., Cuevas M.E., Moraga-Cid G., Fuentealba J., Rivas C.I., Rezaei H., Muñoz-Montesino C. (2021). PrPC as a transducer of physiological and pathological signals. Front. Mol. Neurosci..

[46] Legname G., Scialò C. (2020). On the role of the cellular prion protein in the uptake and signaling of pathological aggregates in neurodegenerative diseases. Prion.

[47] Beraldo F.H., Ostapchenko V.G., Caetano F.A., Guimaraes A.L.S., Ferretti G.D.S., Daude N., Bertram L., Nogueira K.O.P.C., Silva J.L., Westaway D. (2016). Regulation of amyloid β oligomer binding to neurons and neurotoxicity by the prion protein-mGluR5 complex. J. Biol. Chem..

[48] Balducci C., Beeg M., Stravalaci M., Bastone A., Sclip A., Biasini E., Tapella L., Colombo L., Manzoni C., Borsello T. (2010). Synthetic amyloid-β oligomers impair long-term memory independently of cellular prion protein. Proc. Natl. Acad. Sci. USA.

[49] Hasegawa M. (2019). Structure of NFT: Biochemical pproach. Adv. Exp. Med. Biol..

[50] Trejo-Lopez J.A., Yachnis A.T., Prokop S. (2022). Neuropathology of Alzheimer’s disease. Neurotherapeutics.

[51] Nguyen N.M., Cho J., Lee C. (2023). Gut microbiota and Alzheimer’s disease: How to study and apply their relationship. Int. J. Mol. Sci..

[52] Dissanayaka D.M.S., Jayasena V., Rainey-Smith S.R., Martins R.N., Fernando W.M.A.D.B. (2024). The role of diet and gut microbiota in Alzheimer’s disease. Nutrients.

[53] Angelucci F., Cechova K., Amlerova J., Hort J. (2019). Antibiotics, gut microbiota, and Alzheimer’s disease. J. Neuroinflammation.

[54] Tarawneh R., Penhos E. (2022). The gut microbiome and Alzheimer’s disease: Complex and bidirectional interactions. Neurosci. Biobehav. Rev..

[55] Chandra S., Sisodia S.S., Vassar R.J. (2023). The gut microbiome in Alzheimer’s disease: What we know and what remains to be explored. Mol. Neurodegener..

[56] Ferreiro A.L., Choi J., Ryou J., Newcomer E.P., Thompson R., Bollinger R.M., Hall-Moore C., Ndao I.M., Sax L., Benzinger T.L.S. (2023). Gut microbiome composition may be an indicator of preclinical Alzheimer’s disease. Sci. Transl. Med..

[57] Swarbrick S., Wragg N., Ghosh S., Stolzing A. (2019). Systematic review of miRNA as biomarkers in Alzheimer’s disease. Mol. Neurobiol..

[58] Groot M., Lee H. (2020). Sorting mechanisms for microRNAs into extracellular vesicles and their associated diseases. Cells.

[59] Li X., Chen S.-C., Ip J.P.K. (2022). Diverse and composite roles of miRNA in non-neuronal cells and neuronal synapses in Alzheimer’s disease. Biomolecules.

[60] Koh H.S., Lee S., Lee H.J., Min J.-W., Iwatsubo T., Teunissen C.E., Cho H.-J., Ryu J.-H. (2021). Targeting microRNA-485-3p blocks Alzheimer’s disease progression. Int. J. Mol. Sci..

[61] Chen M.-L., Hong C.-G., Yue T., Li H.-M., Duan R., Hu W.-B., Cao J., Wang Z.-X., Chen C.-Y., Hu X.-K. (2021). Inhibition of miR-331-3p and miR-9-5p ameliorates Alzheimer’s disease by enhancing autophagy. Theranostics.

[62] Jiang Y., Bian W., Chen J., Cao X., Dong C., Xiao Y., Xu B., Sun X. (2023). miRNA-137-5p improves spatial memory and cognition in Alzheimer’s mice by targeting ubiquitin-specific peptidase 30. Anim. Models Exp. Med..

[63] Zheng K., Hu F., Zhou Y., Zhang J., Zheng J., Lai C., Xiong W., Cui K., Hu Y.-Z., Han Z.-T. (2021). miR-135a-5p mediates memory and synaptic impairments via the Rock2/Adducin1 signaling pathway in a mouse model of Alzheimer’s disease. Nat. Commun..

[64] Wang R., Chopra N., Nho K., Maloney B., Obukhov A.G., Nelson P.T., Counts S.E., Lahiri D.K. (2022). Human microRNA (miR-20b-5p) modulates Alzheimer’s disease pathways and neuronal function, and a specific polymorphism close to the MIR20B gene influences Alzheimer’s biomarkers. Mol. Psychiatry.

[65] Nagaraj S., Want A., Laskowska-Kaszub K., Fesiuk A., Vaz S., Logarinho E., Wojda U. (2021). Candidate Alzheimer’s disease biomarker miR-483-5p lowers TAU phosphorylation by direct ERK1/2 repression. Int. J. Mol. Sci..

[66] Walgrave H., Balusu S., Snoeck S., Vanden Eynden E., Craessaerts K., Thrupp N., Wolfs L., Horré K., Fourne Y., Ronisz A. (2021). Restoring miR-132 expression rescues adult hippocampal neurogenesis and memory deficits in Alzheimer’s disease. Cell Stem Cell.

[67] Liu Y., Xu Y., Yu M. (2022). MicroRNA-4722-5p and microRNA-615-3p serve as potential biomarkers for Alzheimer’s disease. Exp. Ther. Med..

[68] Dhiman K., Blennow K., Zetterberg H., Martins R.N., Gupta V.B. (2019). Cerebrospinal fluid biomarkers for understanding multiple aspects of Alzheimer’s disease pathogenesis. Cell Mol. Life Sci..

[69] Maltais M., De Souto Barreto P., Hooper C., Payoux P., Rolland Y., Vellas B., MAPT/DSA Study Group (2019). Association between brain β-amyloid and frailty in older adults. J. Gerontol. Ser. A.

[70] Lopes Alves I., Collij L.E., Altomare D., Frisoni G.B., Saint-Aubert L., Payoux P., Kivipelto M., Jessen F., Drzezga A., Leeuwis A. (2020). Quantitative amyloid PET in Alzheimer’s disease: The AMYPAD prognostic and natural history study. Alzheimers Dement..

[71] Pereira J.B., Janelidze S., Stomrud E., Palmqvist S., van Westen D., Dage J.L., Mattsson-Carlgren N., Hansson O. (2021). Plasma markers predict changes in amyloid, tau, atrophy and cognition in non-demented subjects. Brain.

[72] Lu W.-H., Giudici K.V., Rolland Y., Guyonnet S., Li Y., Bateman R.J., de Souto Barreto P., Vellas B. (2021). Prospective associations between plasma amyloid-beta 42/40 and frailty in community-dwelling older adults. J. Prev. Alzheimers Dis..

[73] Peña-Bautista C., Roca M., Hervás D., Cuevas A., López-Cuevas R., Vento M., Baquero M., García-Blanco A., Cháfer-Pericás C. (2019). Plasma metabolomics in early Alzheimer’s disease patients diagnosed with amyloid biomarker. J. Proteomics.

[74] Bergland A.K., Proitsi P., Kirsebom B.-E., Soennesyn H., Hye A., Larsen A.I., Xu J., Legido-Quigley C., Rajendran L., Fladby T. (2020). Exploration of plasma lipids in mild cognitive impairment due to Alzheimer’s disease. J. Alzheimers Dis..

[75] Ashton N.J., Moseby-Knappe M., Benedet A.L., Grötschel L., Lantero-Rodriguez J., Karikari T.K., Hassager C., Wise M.P., Stammet P., Kjaergaard J. (2023). Alzheimer disease blood biomarkers in patients with out-of-hospital cardiac Arrest. JAMA Neurol..

[76] Gill J., Mustapic M., Diaz-Arrastia R., Lange R., Gulyani S., Diehl T., Motamedi V., Osier N., Stern R.A., Kapogiannis D. (2018). Higher exosomal tau, amyloid-beta 42 and IL-10 are associated with mild TBIs and chronic symptoms in military personnel. Brain Inj..

[77] Brett B.L., Gardner R.C., Godbout J., Dams-O’Connor K., Keene C.D. (2022). Traumatic brain injury and risk of neurodegenerative disorder. Biol. Psychiatry.

[78] Prins S., de Kam M.L., Teunissen C.E., Groeneveld G.J. (2022). Inflammatory plasma biomarkers in subjects with preclinical Alzheimer’s disease. Alzheimers Res. Ther..

[79] Chaney A., Williams S.R., Boutin H. (2019). In vivo molecular imaging of neuroinflammation in Alzheimer’s disease. J. Neurochem..

[80] El Idrissi F., Gressier B., Devos D., Belarbi K. (2021). A Computational exploration of the molecular network associated to neuroinflammation in Alzheimer’s disease. Front. Pharmacol..

[81] Carlini V., Verduci I., Cianci F., Cannavale G., Fenoglio C., Galimberti D., Mazzanti M. (2020). CLIC1 Protein Accumulates in Circulating Monocyte Membrane during Neurodegeneration. Int. J. Mol. Sci..

[82] Kenny A., Jiménez-Mateos E.M., Zea-Sevilla M.A., Rábano A., Gili-Manzanaro P., Prehn J.H.M., Henshall D.C., Ávila J., Engel T., Hernández F. (2019). Proteins and microRNAs are differentially expressed in tear fluid from patients with Alzheimer’s disease. Sci. Rep..

[83] Fotuhi S.N., Khalaj-Kondori M., Hoseinpour Feizi M.A., Talebi M. (2019). Long non-coding RNA BACE1-AS may serve as an Alzheimer’s disease blood-based biomarker. J. Mol. Neurosci..

[84] Nie C., Sun Y., Zhen H., Guo M., Ye J., Liu Z., Yang Y., Zhang X. (2020). Differential expression of plasma exo-miRNA in neurodegenerative diseases by next-generation sequencing. Front. Neurosci..

[85] Visconte C., Fenoglio C., Serpente M., Muti P., Sacconi A., Rigoni M., Arighi A., Borracci V., Arcaro M., Arosio B. (2023). Altered extracellular vesicle miRNA profile in prodromal Alzheimer’s disease. Int. J. Mol. Sci..

[86] Liu W.-L., Lin H.-W., Lin M.-R., Yu Y., Liu H.-H., Dai Y.-L., Chen L.-W., Jia W.-W., He X.-J., Li X.-L. (2022). Emerging blood exosome-based biomarkers for preclinical and clinical Alzheimer’s disease: A meta-analysis and systematic review. Neural Regen Res.

[87] Kouhnavardi S., Cabatic M., Mañas-Padilla M.C., Malabanan M.A., Smani T., Cicvaric A., Muñoz Aranzalez E.A., Koenig X., Urban E., Lubec G. (2023). miRNA-132/212 deficiency disrupts selective corticosterone modulation of dorsal vs. ventral hippocampal metaplasticity. Int. J. Mol. Sci..

[88] Chen Y., Gao X., Pei H. (2022). miRNA-384-3p alleviates sevoflurane-induced nerve injury by inhibiting Aak1 kinase in neonatal rats. Brain Behav..

[89] Liu C., Tong Z., Tan J., Xin Z., Wang Z., Tian L. (2019). MicroRNA-21-5p targeting PDCD4 suppresses apoptosis via regulating the PI3K/AKT/FOXO1 signaling pathway in tongue squamous cell carcinoma. Exp. Ther. Med..

[90] Duan X., Zheng Q., Liang L., Zhou L. (2024). Serum exosomal miRNA-125b and miRNA-451a are potential diagnostic biomarker for Alzheimer’s diseases. Degener. Neurol. Neuromuscul. Dis..

[91] Wang J., Chen C., Zhang Y. (2020). An investigation of microRNA-103 and microRNA-107 as potential blood-based biomarkers for disease risk and progression of Alzheimer’s disease. J. Clin. Lab. Anal..

[92] Souza V.C., Morais G.S., Henriques A.D., Machado-Silva W., Perez D.I.V., Brito C.J., Camargos E.F., Moraes C.F., Nóbrega O.T. (2020). Whole-blood levels of microRNA-9 are decreased in patients with late-onset Alzheimer disease. Am. J. Alzheimers Dis. Other Demen..

[93] Li Q., Wang Y., Peng W., Jia Y., Tang J., Li W., Zhang J.H., Yang J. (2019). MicroRNA-101a regulates autophagy phenomenon via the MAPK pathway to modulate Alzheimer’s-associated pathogenesis. Cell Transplant..

[94] Li S., Poon C.H., Zhang Z., Yue M., Chen R., Zhang Y., Hossain M.F., Pan Y., Zhao J., Rong L. (2023). MicroRNA-128 suppresses tau phosphorylation and reduces amyloid-beta accumulation by inhibiting the expression of GSK3β, APPBP2, and mTOR in Alzheimer’s disease. CNS Neurosci. Ther..

[95] Kim S.J., Russell A.E., Wang W., Gemoets D.E., Sarkar S.N., Simpkins J.W., Brown C.M. (2022). miR-146a dysregulates energy metabolism during neuroinflammation. J. Neuroimmune Pharmacol..

[96] Cao Y., Tan X., Lu Q., Huang K., Tang X., He Z. (2021). MiR-29c-3p may promote the progression of Alzheimer’s disease through BACE1. J. Healthc. Eng..

[97] Wang Y., Shi M., Hong Z., Kang J., Pan H., Yan C. (2021). MiR-130a-3p has protective effects in Alzheimer’s disease via targeting DAPK1. Am. J. Alzheimers Dis. Other Dement..

[98] Feng H., Hu P., Chen Y., Sun H., Cai J., He X., Cao Q., Yin M., Zhang Y., Li Q. (2023). Decreased miR-451a in cerebrospinal fluid, a marker for both cognitive impairment and depressive symptoms in Alzheimer’s disease. Theranostics.

[99] Fu L., Jiang G., Weng H., Dick G.M., Chang Y., Kassab G.S. (2019). Cerebrovascular miRNAs correlate with the clearance of Aβ through perivascular route in younger 3xTg-AD mice. Brain Pathol..

[100] Rodriguez-Ortiz C.J., Prieto G.A., Martini A.C., Forner S., Trujillo-Estrada L., LaFerla F.M., Baglietto-Vargas D., Cotman C.W., Kitazawa M. (2020). miR-181a negatively modulates synaptic plasticity in hippocampal cultures and its inhibition rescues memory deficits in a mouse model of Alzheimer’s disease. Aging Cell.

[101] Yao X., Xian X., Fang M., Fan S., Li W. (2020). Loss of miR-369 promotes tau phosphorylation by targeting the Fyn and serine/threonine-protein kinase 2 signaling pathways in Alzheimer’s disease mice. Front. Aging Neurosci..

[102] Hosseinian S., Arefian E., Rakhsh-Khorshid H., Eivani M., Rezayof A., Pezeshk H., Marashi S.-A. (2020). A meta-analysis of gene expression data highlights synaptic dysfunction in the hippocampus of brains with Alzheimer’s disease. Sci. Rep..

[103] Estfanous S., Daily K.P., Eltobgy M., Deems N.P., Anne M.N.K., Krause K., Badr A., Hamilton K., Carafice C., Hegazi A. (2021). Elevated expression of miR-17 in microglia of Alzheimer’s disease patients abrogates autophagy-mediated amyloid-β degradation. Front. Immunol..

[104] Shi Z., Zhang K., Zhou H., Jiang L., Xie B., Wang R., Xia W., Yin Y., Gao Z., Cui D. (2020). Increased miR-34c mediates synaptic deficits by targeting synaptotagmin 1 through ROS-JNK-p53 pathway in Alzheimer’s disease. Aging Cell.

[105] Xiao G., Chen Q., Zhang X. (2021). MicroRNA-455–5p/CPEB1 pathway mediates Aβ-related learning and memory deficits in a mouse model of Alzheimer’s disease. Brain Res. Bull..

[106] Liu C.-G., Zhao Y., Lu Y., Wang P.-C. (2021). ABCA1-labeled exosomes in serum contain higher microRNA-193b levels in Alzheimer’s disease. BioMed Res. Int..

[107] Li Y., Meng S., Di W., Xia M., Dong L., Zhao Y., Ling S., He J., Xue X., Chen X. (2022). Amyloid-β protein and microRNA-384 in NCAM-labeled exosomes from peripheral blood are potential diagnostic markers for Alzheimer’s disease. CNS Neurosci. Ther..

[108] Siedlecki-Wullich D., Miñano-Molina A.J., Rodríguez-Álvarez J. (2021). microRNAs as early biomarkers of Alzheimer’s disease: A synaptic perspective. Cells.

[109] Jaber V.R., Zhao Y., Sharfman N.M., Li W., Lukiw W.J. (2019). Addressing Alzheimer’s disease (AD) neuropathology using anti-microRNA (AM) strategies. Mol. Neurobiol..

